# The Influence of Altitude, Urbanization, and Local Vaccination Centers on Vaccine Uptake within an Italian Health District: An Analysis of 15,000 Individuals Eligible for Vaccination

**DOI:** 10.3390/vaccines12080875

**Published:** 2024-08-02

**Authors:** Andrea Ceccarelli, Giorgia Soro, Chiara Reali, Emilia Biguzzi, Roberta Farneti, Valeria Frassineti, Raffaella Angelini, Gian Luigi Belloli, Davide Gori, Marco Montalti

**Affiliations:** 1 Operative Unit of Hygiene and Public Health—Forlì and Cesena, Department of Public Health, Romagna Local Health Authority, 47522 Cesena, Italy; 2 Unit of Hygiene, Department of Biomedical and Neuromotor Sciences, University of Bologna, 40126 Bologna, Italy; 3 Operative Unit of Hygiene and Public Health-Ravenna, Department of Public Health, Romagna Local Health Authority, 48121 Ravenna, Italy

**Keywords:** vaccine confidence, vaccine coverage, vaccination campaign, barriers, epidemiology

## Abstract

In Italy, free vaccinations for Herpes Zoster (HZ), pneumococcal (PCV), and influenza (FLU) are recommended each year for individuals turning 65. Despite this, achieving optimal vaccination coverage remains challenging. This study assesses coverage rates for HZ, PCV, and FLU in Forlì, Northern Italy, and examines how altitude, urban planning, and health organization variables (such as the presence of a vaccination center) impact vaccine uptake. Vaccination coverages were calculated for birth cohorts between 1952 and 1958 for each municipality in the Forlì area as of 1 March 2024. The geographical factors influencing the vaccination uptake were extracted from the Italian National Institute of Statistics (ISTAT) records and evaluated through a multivariate analysis. The sample analyzed included 15,272 vaccine campaign targets from Forlì’s province (185,525 citizens); the vaccine uptake rates for HZ, PCV, and FLU were 26.9%, 36.7%, and 43.5%, respectively. Gender did not appear to influence vaccine uptake. Living in a flat area appeared to increase vaccine uptake in a statistically significant way for all the vaccinations when compared to a mountainous area (HZ: OR: 1.50, PCV: OR: 1.33, FLU: OR: 1.67). The presence of a vaccine service in low-urbanized areas was shown to increase vaccine uptake for all vaccinations (HZ: OR: 1.65, PCV: OR: 1.93, FLU: OR: 1.53) compared with low-urbanized areas without a vaccination center or more urbanized areas with a vaccination center. This study emphasizes the significance of the territorial context, along with the ease of access to vaccinations and geographic barriers, as key determinants in achieving vaccination targets. Local health authorities should consider these factors when implementing vaccination campaigns.

## 1. Introduction

**Figure 1:**
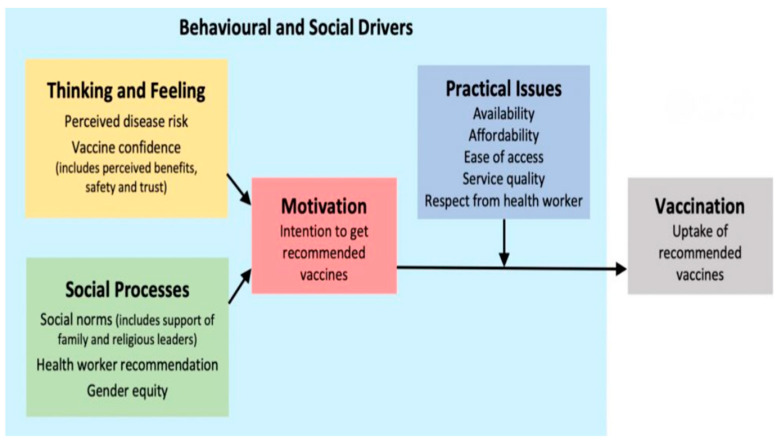
Behavioral and social drivers of vaccination framework. From https://iris.who.int/bitstream/handle/10665/354458/WER9720-eng-fre.pdf (accessed on 30 July 2024). Source: The BeSD working group. Based on Brewer et al., 2017 [8].

The Italian Ministry of Health through the national immunization plan provides and recommends vaccinations for influenza (FLU), pneumococcal (PCV), and shingles (HZ) for several categories of patients at risk of medical conditions or birth cohorts by virtue of their increased risk of infection [1]. At the regional level, the national plan is adopted and disseminated through the regional preventive vaccination plan (PRPV), which can implement the vaccination supply for its residents. Ministerial and regional long-term target vaccination coverage goals for these vaccinations in individuals over 65 years old are higher than 50% for HZ and higher than 75% for FLU and PCV [1,2]. Despite these targets, vaccination coverage remains suboptimal with the Italian average vaccination coverage for FLU in older patients standing at only 57% for the 2022/2023 epidemic season [3]. Italian studies conducted in Italy in 2023 reported that only 40% of the adult vaccine target population had received the PCV vaccination in their life [4] and only 10% had received the HZ vaccine [5]. This significant distance from the set ministerial targets requires health authorities to better understand patient concerns, behaviors, and social determinants regarding these vaccinations to implement strategies to improve vaccination coverage. To support national and local vaccine programs and the systematic assessment of factors affecting uptake, the World Health Organization (WHO) has developed a tool called “Measuring Behavioural and Social Drivers of Vaccination” (BeSD) [6]. The “BeSD” vaccination model (Figure 1) is a conceptual framework for mapping the relationship between vaccine confidence, motivation, behavior, and practical issues that can be helpful in elucidating the vaccination attitudes of a population [6,7].

In particular, the influence of practical issues was confirmed by many studies both at the Italian and international levels. Italian studies assessing the perceived ease of accessing healthcare facilities for HZ, PCV, or FLU vaccines have shown that the nationwide pattern is patchy. The perceived levels of difficulty in accessing vaccinations were rated as “medium” or “high”, with higher percentages in the islands (HZ: 27.9%, PCV: 25.6%, FLU: 18.3%) compared to northeastern Italy (HZ: 14.1%, PCV: 14.5%, FLU: 13.0%) [4,5,7]. Also, internationally, studies conducted in both Africa and Australia have shown the importance of practical factors in “access to vaccinations”. In an interesting study conducted in Africa in 2022 involving 27,240 healthy workers, access was highlighted as a crucial barrier, with less than a quarter reporting that accessing SARS-CoV-2 vaccination services for themselves would have been very easy [9]. Moreover, in a 2023 Australian study in which older adults over 70 years old were surveyed, factors related to ease of access were identified as important proxies in the decision to vaccinate or not for SARS-CoV-2 [10].

In addition to ease of access, other practical geographic factors (e.g., population density, level of urbanization, distance from vaccination centers) have also been investigated in the literature to determine their link to vaccination uptake. A 2015 study aimed at highlighting the link between certain socio-demographic variables and rotavirus vaccine uptake in children underlined how administrative units with higher population densities generally have lower vaccination rates than less populated ones [11]. This finding was also confirmed by a Canadian study regarding the acceptance of flu vaccination, where fewer vaccinations took place at centers located in areas with high residential density rates [12]. Finally, an Argentine study from 2024, in which adolescents accessing a healthcare center in Buenos Aires were surveyed, found that the “distance from vaccination centers” emerged as one of the reasons for non-vaccination or incomplete vaccination schedules [13]. In a systematic review of the literature regarding barriers to vaccination access in Latin America, “geographic barriers” were identified as one of the most common determinants, mentioned in 7% of all the reviewed articles [14].

All of these literature findings underscore the importance for local health authorities to comprehend the prevalence of these practical issues within their jurisdiction. The objective of this study was to assess how altitude, urbanization, and the presence of local vaccination centers influence vaccine uptake for HZ, PCV, and FLU among the population residing in Forlì, Italy, aiming to guide targeted actions by the local health authorities.

## 2. Materials and Methods

This retrospective observational study examined all recipients of FLU, PCV, and HZ vaccines born between 1952 and 1958, residing in the Forlì health district. Forlì is one of eight health districts within the Romagna Local Health Authority (LHA). The Forlì health district spans approximately 228.20 square kilometers and has a population of 117,210 individuals, encompassing fifteen municipalities: Bertinoro, Castrocaro Terme e Terra del Sole, Civitella di Romagna, Dovadola, Forlì, Forlimpopoli, Galeata, Meldola, Modigliana, Portico e San Benedetto, Predappio, Premilcuore, Rocca San Casciano, Santa Sofia, and Tredozio.

### 2.1. Data Extraction and Collection

Vaccination data were extracted from the LHA vaccination registry on 1 March 2024, and coverage data are considered up-to-date as of that date. The LHA vaccination registry is updated simultaneously with each vaccination and is continuously maintained, ensuring access to comprehensive information on all vaccinations administered to LHA patients. From the vaccination registry, the vaccination status (vaccinated or not vaccinated) of each patient was extracted for each vaccine included in the study (FLU, PCV, and HZ). Additionally, information on the year of birth, gender of the recipient, and municipality of residence was also obtained.

The classification of each municipality by altitude and urbanization area was sourced from the records of the Italian National Institute of Statistics (ISTAT). According to ISTAT, the altitude zones of the municipalities are divided into mountain zones (elevations above 600/700 m above sea level), hill zones (elevations between 300 and 600 m above sea level), and plain zones (land not exceeding 300 m above sea level) [15]. The level of urbanization is also categorized into three types of municipalities: “Cities” or “Densely populated areas”, “Small towns and suburbs” or “Intermediate population density areas”, and “Rural areas” or “Low populated areas” [16].

### 2.2. Statistical Analysis

Variables were characterized using both absolute frequencies and percentages. Gender, municipality of residence, altitude of the municipality of residence, level of urbanization, and the presence of a vaccination center in the municipality were evaluated as factors potentially influencing vaccine uptake through a multivariate analysis. Specifically, a composite variable named “level of urbanization and presence of vaccination center in the municipalities” was created, consisting of the factors “level of urbanization” and “presence of vaccination center in the municipality”.

The results of the multivariate analyses are displayed as odds ratios (ORs) accompanied by their standard errors (SEs) and a 95% confidence interval (C.I.). The threshold for statistical significance was set at *p* < 0.05. All statistical analyses were executed using Stata Statistical Software 15, developed by StataCorp., College Station, TX, USA.

## 3. Results

### 3.1. Main Sample Features

Table 1 presents a comprehensive overview of the study sample’s main characteristics, consisting of a total of 15,272 individuals. Participants had the following distribution with respect to their year of birth: 1952 (n = 2011; 13.2%), 1953 (n = 2069; 13.6%), 1954 (n = 2075; 13.6%), 1955 (n = 2267; 14.8%), 1956 (n = 2272; 14.9%), 1957 (n = 2222; 14.6%), and 1958 (n = 2356; 15.4%). The gender distribution showed slight variations, with female participants comprising 53.8%. Regarding vaccination uptake, the distribution was the following: HZ (n = 4106; 26.9%), PCV (n = 5610; 36.7%), and FLU (n = 6649; 43.5%). When exploring the altitude zone of the municipality of residence, the majority of the population lived in flat areas (n = 11,447; 75.0%), while the remaining part lived in hilly and mountainous areas, respectively (n = 3323; 21.8%; n = 502; 3.3%). A larger part of the studied population lived in densely populated areas (n = 9553; 62.6%) compared to those living in intermediate and low-density areas (n = 1895; 12.4%; n = 3824; 25.0%). Finally, most of the examined population lived in a municipality with a vaccination center (n = 12,112; 79.3%).

### 3.2. Multivariate Analysis

Table 2 illustrates the factors impacting the uptake of HZ vaccination. A notable gradient in vaccination uptake was observed across birth cohorts from 1953 (OR: 4.92, SE: 0.38, *p* = 0.001, 95% C.I.: 4.23–5.73) to 1958 (OR: 1.31, SE: 0.11, *p* = 0.001, 95% C.I.: 1.11–1.54) when compared to individuals born in 1952, the inaugural year of the national campaign. Gender did not appear to influence vaccine uptake; living in a flat area appeared to increase vaccine uptake in a statistically significant way (OR: 1.50, SE: 0.22, *p* = 0.005, 95% C.I.: 1.13–1.99) when compared to living in a mountainous area. Living in a low-urbanized area with the presence of a local vaccination center statistically significantly increased vaccination for HZ compared with areas without a vaccination center (OR: 1.65, SE: 0.13, *p* = 0.001, 95% C.I.: 1.40–1.93). Additionally, residing in an intermediate-urbanized area with access to a local vaccination center was associated with a statistically significant increase in vaccine uptake compared to areas without such centers, albeit with a lower odds ratio (OR: 1.27, SE: 0.13, *p* = 0.023, 95% C.I.: 1.03–1.56).

Table 3 presents the factors influencing the adoption of PCV vaccination. A significant gradient in vaccination uptake was noted among birth cohorts from 1955 (OR: 1.22, SE: 0.75, *p* = 0.002, 95% C.I.: 1.08–1.37) to 1958 (OR: 0.24, SE: 0.17, *p* = 0.001, 95% C.I.: 0.21–0.27) relative to those born in 1952, the initial year of the national campaign. Gender did not appear to influence vaccine uptake; living in a flat area appeared to increase vaccine uptake in a statistically significant way (OR: 1.33, SE: 0.18, *p* = 0.031, 95% C.I.: 1.03–1.74). Living in a low-, intermediate-, or densely urbanized area with the presence of a local vaccination center statistically significantly increased vaccination for PCV compared with areas without a vaccination center (OR: 1.93, SE: 0.14, *p* = 0.001, 95% C.I.: 1.67–2.23), (OR: 1.30, SE: 0.13, *p* = 0.009, 95% C.I.: 1.07–1.58), (OR: 1.24, SE: 0.97, *p* = 0.007, 95% C.I.: 1.06–1.44), respectively.

Table 4 reveals the factors impacting FLU vaccination uptake. All cohorts from 1954 (OR: 0.83, SE: 0.52, *p* = 0.002, 95% C.I.: 0.73–0.93) to 1958 (OR: 0.42, SE: 0.26, *p* = 0.001, 95% C.I.: 0.37–0.47) exhibited statistically significant odds ratios indicating lower FLU vaccine uptake compared to those born in 1952. Gender did not appear to influence vaccine uptake; living in a flat area appeared to increase vaccine uptake in a statistically significant way (OR: 1.67, SE: 0.21, *p* = 0.001, 95% C.I.: 1.31–2.14). Living in a low-urbanized area with the presence of a local vaccination center statistically significantly increased vaccination for FLU compared with areas without a vaccination center (OR: 1.53, SE: 0.11, *p* = 0.001, 95% C.I.: 1.33–1.76).

## 4. Discussion

This study conducted in Forlì, Northern Italy, has shown that PCV and FLU vaccines have higher acceptance rates (PCV: 36.7%, FLU: 43.5%) compared to HZ vaccination, which had an acceptance rate of 26.9%. The presence of a vaccination center in less urbanized areas significantly increased the probability of vaccine uptake for all vaccinations (HZ: +65%, PCV: +93%, FLU: +53%) compared to areas without a vaccination center or in more urbanized areas that have a vaccination center. Across all three vaccinations reviewed, uptake tended to be higher among older people than younger people within the analyzed age groups. Living in a lowland area rather than a mountainous area was found to increase vaccine acceptance for all the vaccinations. The higher uptake for PCV and FLU compared to HZ is consistent with findings from the OBVIOUS project, a series of cross-sectional studies of the Italian population in 2022. In these studies, 45.4% and 39.5% of respondents reported being vaccinated for FLU and PCV, respectively, whereas only 9.6% reported being vaccinated for HZ on average at the national level [4,5,7].

For all three vaccinations, coverage remains suboptimal. However, the feasibility of administering PCV and FLU vaccinations at general practitioner (GP) offices may contribute to their higher acceptance rates. This hypothesis may be supported by a 2023 Chinese randomized controlled study, where the active engagement of GPs from a community health center resulted in increased vaccination rates among patients (1100 individuals) and a 24.7% improvement in patient awareness of FLU vaccination compared to the previous season [17]. Emerging evidence indicates a need to implement strategies aimed at increasing vaccine awareness and uptake among elderly patients for HZ. This recommendation is bolstered by observational studies, including a 2024 Italian study, which found that individuals aged 74 years and older faced approximately three times the risk of prolonged hospitalization due to HZ compared to younger patients [18].

For each of the three vaccinations under investigation, it was consistently observed that vaccine acceptance rates were higher among older individuals compared to younger ones within the studied age groups. This observation aligns with the policy in Italy in effect since 2017, where all examined vaccinations are provided free of charge starting at age 65, with this entitlement continuing throughout life. Therefore, older citizens have had more opportunities to access these vaccinations over time.

Currently, there is limited substantial evidence concerning the impact of practical determinants and geographic barriers on vaccine acceptance in the Italian context. However, our study has demonstrated that residing in lowland areas was statistically associated with higher vaccine uptake compared to mountainous areas for all analyzed vaccinations. This observation finds support in the literature, exemplified by a 2022 study on SARS-CoV-2 vaccine uptake among U.S. children, which noted notably lower uptake rates in mountainous states (e.g., Wyoming at 53.5%) compared to states in the New England area (e.g., Massachusetts at 89.4%) [19]. The reasons for these disparities may be manifold. A study conducted in the United States, examining socio-behavioral drivers influencing SARS-CoV-2 vaccination among U.S. adults, found that unvaccinated individuals residing in the Mountain West region generally exhibited lower risk perception (median = 15.0%), less confidence in vaccine safety (median = 12.0%), and lower perceived importance of vaccination (median = 12.0%) compared to their counterparts in New York/New Jersey, where unvaccinated individuals showed higher risk perception (median = 26.5%), greater confidence in vaccine safety (median = 15.4%), and higher perceived importance of vaccination (median = 31.2%) [20].

For all three vaccinations investigated, the multivariate analysis indicated that residing in a sparsely populated area with a vaccination center in the municipality significantly increased vaccine uptake compared to areas without a vaccination center. Importantly, the increase in uptake for those living in low-populated areas with a vaccination center was greater than for those living in densely populated areas with a vaccination center, in both cases compared to individuals residing in areas without a vaccination center. The finding regarding the positive influence of proximity to a vaccination center on vaccine uptake is supported by multiple studies in the literature. For example, a 2022 article focused on analyzing the primary drivers of HPV vaccine hesitancy in Malawi identified distance and logistical challenges in accessing health centers as significant barriers to completing pediatric vaccine schedules [21]. Similarly, a cross-sectional survey conducted among caregivers of pediatric patients during the SARS-CoV-2 pandemic found that children whose caregivers found it moderately easy (aOR: 2.26, 95% C.I.: 1.06–4.83) or very easy (aOR: 2.22, 95% C.I.: 1.06–4.69) to access vaccination centers had higher odds of being fully vaccinated for their age compared to children whose caregivers found it difficult or somewhat easy to access vaccination centers [22]. However, what appears particularly noteworthy from our analysis is that the influence of local vaccination centers remained significant regardless of the population density of the municipality in question; in fact, it appeared to be more pronounced in less densely populated communities. This phenomenon could be linked to a stronger sense of community in smaller municipalities, as supported by a study involving a sample of 2490 Americans on the impact of prosocial concerns on FLU vaccination. The study revealed that prosocial concerns significantly promoted earlier vaccination in regions with lower population density (z = −2.89, *p* = 0.004, 95% C.I.: −0.15, −0.03) [23]. Another contributing factor that could help explain this result is “word-of-mouth”, which in small suburban centers could serve as a crucial source of information to reach a broader audience. This phenomenon was also highlighted in research conducted by B. Friedman et al. regarding barriers to recruiting patients for scientific studies [24]. The influence of word-of-mouth on public healthcare initiatives has also been underscored in a retrospective multicenter study conducted in 2024 across four vaccination centers (Forlì, Cesena, Ravenna, and Rimini) within the Romagna LHA. In the study, concerning the catch-up vaccination campaign for HZ, word-of-mouth from family and friends emerged as the second primary source of information through which individuals learned about the campaign, surpassing even advice from their GPs [25].

All of the determinants and practical issues analyzed in this paper are crucial for structuring future vaccination campaigns tailored to specific territorial contexts. This conclusion seems to be supported by two recent studies published in 2023 by Kimberly E. Bonner et al. and Calvince Otieno Anino et al., which examined the influence of various factors on the willingness to be vaccinated for FLU and COVID-19. Both studies emphasized that the ease of access to drop-in vaccination sites had the greatest impact on vaccine willingness across all age groups [26,27]. Additionally, another retrospective analysis of factors associated with increased vaccination coverage of children in Senegal, conducted between 2005 and 2019, showed that place of residence, ease of access, and urban density were all key determinants of uptake [28].

In the specific context of this study, in light of the results obtained, practical measures could certainly be implemented to increase ease of access. For example, introducing local vaccination clinics, especially in areas with lower vaccination uptake, could be beneficial. Furthermore, it may be appropriate to conduct active call campaigns targeting cohorts that currently have lower vaccination coverage.

While the findings of this study provide valuable insights into the specific context and population under investigation, certain limitations must be acknowledged regarding external generalizability. The sample, predominantly drawn from a specific geographic region and demographic profile, may restrict the broader applicability of the results to more diverse populations. Additionally, during the SARS-CoV-2 pandemic in 2020, vaccinations for HZ and PCV were temporarily suspended and resumed in subsequent years, potentially impacting overall vaccine uptake during those periods. Despite these limitations, our findings suggest that implementing more vaccination centers in local areas or expanding vaccine access points (e.g., at GP centers) could be effective strategies to achieve target vaccine uptake levels regardless of urbanization level. Further studies and insights would be beneficial to better understand these and other vaccine determinants related to access difficulties and geographic barriers. An annual follow-up study could be particularly useful to observe whether the actions taken to increase coverage have been effective in altering current outcomes.

## 5. Conclusions

In conclusion, our study underscores the significance of territorial factors such as altitude and urbanization, alongside health organizational elements like the proximity of vaccination centers, as pivotal considerations in designing effective vaccination campaigns. It highlights the need for targeted cohort recall strategies, particularly aimed at populations in mountainous regions, and advocates for the establishment of vaccination centers in underserved areas, prioritizing municipalities with low population density. The findings of this study call for continued investigation and suggest potential pathways for developing tailored vaccination strategies by local health authorities. These strategies could enhance vaccine accessibility and uptake, thereby contributing to improved public health outcomes.

## Figures and Tables

**Table 1:** Main characteristics of the study sample (n = 15,272).

Variable	Modality	N (%)
Municipalities	Bertinoro	839 (5.5)
	Castrocaro Terme e Terra del Sole	561 (3.7)
	Civitella di Romagna	306 (2.0)
	Dovadola	148 (1.0)
	Forlì	9553 (62.6)
	Forlimpopoli	1005 (6.9)
	Galeata	205 (1.3)
	Meldola	840 (5.5)
	Modigliana	427 (2.8)
	Portico e San Benedetto	58 (0.4)
	Predappio	534 (3.5)
	Premilcuore	79 (0.5)
	Rocca San Casciano	178 (1.2)
	Santa Sofia	365 (2.4)
	Tredozio	124 (0.8)
Cohort of birth	1952	2011 (13.2)
	1953	2069 (13.6)
	1954	2075 (13.6)
	1955	2267 (14.8)
	1956	2272 (14.9)
	1957	2222 (14.6)
	1958	2356 (15.4)
Gender	Male	7055 (46.2)
	Female	8217 (53.8)
Vaccination uptake	Shingles	4106 (26.9)
	Pneumococcal	5610 (36.7)
	FLU	6649 (43.5)
Altitude zone of the municipality of residence	Mountain	502 (3.3)
	Hill	3323 (21.8)
	Lowland	11,447 (75.0)
Level of urbanization	Densely populated area	9553 (62.6)
	Intermediate populated area	1895 (12.4)
	Lowly populated area	3824 (25.0)
Presence of vaccination center in own municipality	Yes	12,112 (79.3)
	No	3160 (20.7)

**Table 2:** Variables influencing the HZ vaccination uptake (statistically significant *p*-Values are indicated in bold).

Vaccination against HZ		OR	SE	*p*-Value	95% C.I.
Cohort of birth	1952	1			
	1953	4.92	0.38	**<0.001**	4.23–5.73
	1954	3.80	0.30	**<0.001**	3.26–4.43
	1955	2.12	0.17	**<0.001**	1.81–2.48
	1956	1.86	0.15	**<0.001**	1.59–2.18
	1957	1.85	0.15	**<0.001**	1.58–2.17
	1958	1.31	0.11	**<0.001**	1.11–1.54
Gender	Male	1			
	Female	1.05	0.40	0.197	0.98–1.13
Altitude zone of the municipality of residence	Mountain	1			
	Hill	0.98	0.11	0.860	0.78–1.23
	Lowland	1.50	0.22	**0.005**	1.13–1.99
Level of urbanization and presence of a vaccination center in the municipalities	No presence of a vaccination center in the municipalities	1			
	Densely urbanized area and presence of a vaccination center in the municipalities	1.02	0.84	0.829	0.87–1.20
	Intermediate-urbanized area and presence of a vaccination center in the municipalities	1.27	0.13	**0.023**	1.03–1.56
	Low-urbanized area and presence of a vaccination center in the municipalities	1.65	0.13	**<0.001**	1.40–1.93

**Table 3:** Variables influencing the PCV vaccination uptake (statistically significant *p*-Values are indicated in bold).

Vaccination against PCV		OR	SE	*p*-Value	95% C.I.
Cohort of birth	1952	1			
	1953	0.99	0.63	0.919	0.88–1.12
	1954	1.13	0.71	0.054	1.00–1.28
	1955	1.22	0.75	**0.002**	1.08–1.37
	1956	0.48	0.31	**<0.001**	0.42–0.55
	1957	0.51	0.33	**<0.001**	0.45–0.58
	1958	0.24	0.17	**<0.001**	0.21–0.27
Gender	Male	1			
	Female	1.03	0.36	0.476	0.96–1.10
Altitude zone of the municipality of residence	Mountain	1			
	Hill	1.06	0.11	0.591	0.86–1.30
	Lowland	1.33	0.18	**0.031**	1.03–1.74
Level of urbanization and presence of a vaccination center in the municipalities	No presence of a vaccination center in the municipalities	1			
	Densely urbanized area and presence of a vaccination center in the municipalities	1.23	0.97	**0.007**	1.06–1.44
	Intermediate urbanized area and presence of a vaccination center in the municipalities	1.30	0.13	**0.009**	1.07–1.58
	Low urbanized area and presence of a vaccination center in the municipalities	1.93	0.14	**<0.001**	1.67–2.23

**Table 4:** Variables influencing the FLU vaccination uptake (statistically significant *p*-Values are indicated in bold).

Vaccination against FLU		OR	SE	*p*-Value	95% C.I.
Cohort of birth	1952	1			
	1953	0.92	0.58	0.185	0.81–1.04
	1954	0.82	0.52	**0.002**	0.73–0.93
	1955	0.69	0.43	**0.001**	0.62–0.78
	1956	0.61	0.38	**<0.001**	0.54–0.68
	1957	0.56	0.35	**<0.001**	0.49–0.63
	1958	0.42	0.26	**<0.001**	0.37–0.47
Gender	Man	1			
	Female	1.00	0.33	0.966	0.94–1.07
Altitude zone of the municipality of residence	Mountain	1			
	Hill	0.99	0.10	0.910	0.81–1.21
	Lowland	1.67	0.21	**<0.001**	1.31–2.14
Level of urbanization and presence of a vaccination center in the municipalities	No presence of a vaccination center in the municipalities	1			
	Densely urbanized area and presence of a vaccination center in the municipalities	0.83	0.61	**0.013**	0.72–0.96
	Intermediate urbanized area and presence of a vaccination center in the municipalities	0.79	0.74	**0.012**	0.66–0.95
	Low urbanized area and presence of a vaccination center in the municipalities	1.53	0.11	**<0.001**	1.33–1.76

## Data Availability

The data presented in this study are available upon request from the corresponding author.
